# Re‐defining and tackling the emerging challenges in stem cell research and translation: A report of the 10th CSSCR annual meeting

**DOI:** 10.1111/cpr.13186

**Published:** 2022-03-18

**Authors:** Jingyi Cao, Baoyang Hu, Weizhi Ji

**Affiliations:** ^1^ Institute of Zoology Chinese Academy of Sciences Beijing China; ^2^ Institute for Stem Cell and Regenerative Medicine Chinese Academy of Sciences Beijing China; ^3^ Beijing Institute for Stem Cell and Regenerative Medicine Beijing China; ^4^ Chinese Society for Stem Cell Research Branch Society of Chinese Society of Cell Biology Beijing China; ^5^ State Key Laboratory of Primate Biomedical Research Institute of Primate Translational Medicine Kunming University of Science and Technology Kunming China

## Abstract

The 10th congress of the Chinese Society for Stem Cell Research was held from 11 to 13 October 2020 at Guiyang International Eco‐Conference Center in Guizhou (Southwest China) with 700 offline participants (and 500 online participants). With a theme adopted from a poem by Mao Zedong during the long march, ‘However difficult it might appear; the challenges will be overcome’, the congress provided opportunities for the fast‐growing field to exchange ideas among people from academia, industry and regulatory authorities, to help accelerate translation. Eight plenary lectures and six concurrent sessions on cell fate decision, stem cell metabolism, neural stem cells and neural regeneration, organoids and disease models, tissue and organ regeneration and clinical translation of stem cell research were covered, including 77 oral presentations and 75 poster presentations. The congress also included special programmes of a youth forum, a lecture award programme, a flagship journal forum and a dedicated networking session. This 3‐day event will significantly boost the stem cell research in an era closing to application.

## 1 SIGNIFICANCE STATEMENT

Chinese Society for Stem Cell Research (CSSCR) was established in 2007, as the branch society of the Chinese Society for Cell Biology. The CSSCR is now the largest society for stem cell and regenerative medicine research in China, with 53 directors and over 2000 registered members from all over China. CSSCR holds an annual conference at different locations across China. The participants come from various domains of academia, clinic, industry and government. Responsibilities of the CSSCR include promoting academic exchanges, popularizing science education, developing international cooperation, recommending outstanding talents and advising on policymaking in the field of stem cell research.

## 2 OVERARCHING THEME

The theme of the congress was ‘However difficult it might appear; the challenges will be overcome’. This theme was adopted from a poem written by Mao Zedong in February 1935, during the long march at Loushan Pass in Guizhou province (southwest China), to echo where the annual congress was held this year. The conference was aimed to deliver a message of ‘re‐defining and tackling the hurdles in translational research and clinical application’.

## 3 OBJECTIVES

According to the mission of CSSCR and the theme of this congress, the event also aimed to provide opportunities and a platform for people from academia, industry and regulatory authorities to share advances and accelerate the translation of stem cell research. Concurrent sessions on classical topics such as cell fate decisions, stem cell metabolism, organoids, disease models and those on organ regeneration and clinical applications were planned, in line with the theme ‘However difficult it might appear; the challenges will be overcome’. We expect people will benefit from talks, posters, special events and direct communication among peers. This congress will also has a significant and ongoing impact on regulatory authorities and policymakers of stem cell research and translation.

## 4 PARTICIPANTS

As we are experiencing unprecedented challenges caused by the COVID‐19 pandemic, the congress was organized to bring together traditional in‐person meetings and livestream teleconferencing, to give delegates both offline and online options. A total of 1200 participants, including 700 offline and 500 online participants, attended the scientific concurrent sessions.

## 5 KEYNOTE AND PLENARY LECTURES

The congress provided four plenary lectures on Day 1 and another four on Day 3. The first 4 lectures were co‐chaired by Zhixu He and Xin Xie. Prof Tao Cheng, vice president of CSSCR and Director of the Institute of Hematology & Blood Disease Hospital, Chinese Academy of Medical Sciences, delivered a keynote lecture entitled ‘Understanding How Stem Cells Fit in a Damaged Microenvironment’. He systematically elaborated mechanisms that underlie the regulation of hematopoietic stem cells and their physical microenvironment and summarized the basic requirements for establishing stem cell therapeutic approaches. He emphasized the importance of lineage tracing and biobanking of hematopoietic stem cells (HSCs) for scientific discovery and industry development. He also mentioned that the powerful platform of HSC transplantation will facilitate R&D of cell and gene therapies. Prof Lin Liu from Nankai University talked on ‘Genome Stable Pluripotent Stem Cells and Their Application in Oocyte Regeneration’, focusing on retrotransposons that act on the dynamic pluripotency of embryonic stem cells, oocyte generation from genome stable naïve pluripotent stem cells and oocyte rejuvenation from parthenogenetic stem cells. Prof Jianwu Dai from Institute of Genetics and Developmental Biology, Chinese Academy of Sciences delivered a lecture entitled ‘Progress in Spinal Cord Injury Regeneration’. From a translational aspect, he summarized the advances on microenvironment reconstruction and spinal cord regeneration, with emphasis on animal model and clinical studies. He also analysed the technical challenges to combine stem cells and smart biomaterial for treatment of spinal cord injury. Stem cell‐based treatment on COVID‐19 was covered comprehensively at the meeting. Prof Ronghua Jin, President of Beijing You'an Hospital presented a lecture on ‘Stem Cell Therapy for COVID‐19 Pneumonia’. Prof Jin shared the latest findings from clinical trials using human embryonic stem cell‐derived anti‐inflammatory cells to treat COVID‐19‐associated acute respiratory distress syndrome. In collaboration with team from the Institute for Stem Cell and Regeneration, Chinese Academy of Sciences, they showed preliminary results of significant improvement and satisfactory outcomes of the cell infusion. This was extremely encouraging in consideration of the global pandemic of COVID‐19.

On the last day of the congress, right before the closing ceremony, four plenary lectures were arranged. Prof Tianqing Li from Kunming University of Science and Technology presented ‘Dissecting Human Peri‐implantation Embryo Development by *in vitro* 3D culture and Stem Cell Models’. Prof Li shared their breakthroughs of using a novel 3D culture system to cultivate human blastocyst up to the primitive streak anlage stage. His group had constructed human complete embryo‐like structures that can model human implantation embryo development. Prof Zibing Jin from Beijing Tongren Hospital and Beijing Institute of Ophthalmology presented on ‘iPSC‐derived Retinal Organoids: Technological Progress and Clinical Applications’. His talk covers several correlated topics such as modelling retinal blastoma (RB) with hESC‐derived organoids, transplantation of human iPSC‐derived RPE and neural progenitors, primate models for retinitis pigmentosa and macular degeneration and co‐culture of pluripotent stem cell‐derived microglia and retinal organoids. Prof Renjie Chai from the Southeast University presented on ‘Construction Studies of Novel Integrative Technical Systems Using Neural Stem Cell Transplantation and Artificial Cochlea’, focusing on using biomaterials to induce neural stem cell differentiation into functional neurons. Academician Xiuwu Bian performed the last keynote speech on ‘Pathological Studies on COVID‐19’. Prof Bian shared their major autopsy pathological findings from patients who succumbed to COVID‐19. The intensive pathological diagnosis and research at organ, tissue, cell, ultra‐structure and molecular levels and shared data on SARS‐CoV‐2 (COVID‐19) extensive pulmonary lesions. For elder patients with primary health conditions, Prof Bian also described the SARS‐CoV‐2 causative effect for multiple organ dysfunction syndrome. These findings provided evidence for specifications in the diagnosis and treatment of COVID‐19 infection.^1^

## 6 CONCURRENT SESSIONS

The congress provided concurrent sessions covering the following 6 aspects: cell fate decision, stem cell metabolism, neural stem cells and neural regeneration, organoids and disease models, tissue and organ regeneration and clinical translation of stem cell research. Altogether, there were a total of 6 concurrent sessions comprising 54 oral presentations. The presentation titles and speakers are listed in Table 1 (see pg. 7 of this document). In line with the theme of this congress, the concurrent session on clinical translation of stem cell research provided presentations on stem cell quality assurance, safety and efficacy evaluation of clinical studies, standards development, as well as stem cell ethics and regulatory policies. The concurrent session on tissue and organ regeneration shared the latest progress of projects supported by the strategic priority research programme of the Chinese Academy of Sciences. The concurrent session on organoids and disease models presented recent work on 3D culture and organoid models in the frontiers of stem cell therapy.

**TABLE 1:** Presentations held in concurrent sessions

Title	Speaker	Affiliation
Concurrent Session 1 Cell Fate Decision		
Co‐chair: Yizeng, Bing Liu		
Chromatin and transcriptional regulation in early embryo development	Wei Jie	Tsinghua University
Mechanic forces drive the first cell‐fate decision of mouse pre‐implantation embryo development	Aibin He	Peking University
Mechanism and clinical study from pluripotent stem cell to platelet	Jiaxi Zhou	Chinese Academy of Medical Sciences
New wine in an old bottle: pivotal roles of snoRNAs in maintaining hematopoietic stem cell self‐renewal	Pengxu Qian	Zhejiang University
Progressive pulmonary fibrosis induced by impaired alveolar regeneration	Tan Tang	National Institute of Biological Sciences, Beijing
Intestinal stem cells go necroptosis against genome instability	Wei Mo	Xiamen University
Mediator‐Ing lineage‐specific transcription and diseases	Gang Wang	Fudan University
Concurrent Session 2 Stem Cell Metabolism		
Chair: Shiqiang Huang		
Post‐transcriptional gene regulation by RNA modification	Yungui Yang	Beijing Institute of Genomics, Chinese Academy of Sciences
Production of functional β cells from embryonic stem cell‐derived islet‐like organoid	Weida Li	Tongji University
Metabolic regulation in stem cell ageing and differentiation	Hongbo Zhang	Sun Yat‐sen University
Metabolic regulation in pluripotency	Tongbiao Zhao	Institute of Zoology, Chinese Academy of Sciences
Induction of mouse fibroblast to inner ear hair cells	Weiqiang Gao	Shanghai Jiao Tong University
Protein phosphatase links insulin signalling to glucose clearance and glycogen synthesis	Li Xu	Tsinghua University
Metabolic regulation in pluripotent stem cells and early embryo development	Jin Zhang	Zhejiang University
Screening and functional studies of novel IncRNA in stem cell genomic stability control	Ping Zheng	Kunming Institute of Zoology, Chinese Academy of Sciences
Glis1 facilitates induction of pluripotency via an epigenome–metabolome–epigenome signalling cascade	Keshi Chen	Guangzhou Institutes of Biomedicine and Health, Chinese Academy of Sciences
Modelling human Nonalcoholic Fatty Liver Disease (NAFLD) with an Organoids‐on‐a‐chip System	Yaqing Wang	Dalian Institute of Chemical Physics, Chinese Academy of Sciences
Concurrent Session 3 Neural Stem Cells and Neural Regeneration		
Co‐chair: Jianwei Jiao, Tian Xue		
Human retina and retinal organoid	Tian Xue	University of Science and Technology of China
Mechanism of brain development and neural stem cell	Jianwei Jiao	Institute of Zoology, Chinese Academy of Sciences
Restoration of FMRP expression at puberty rescues visual processing disorders in a mouse model of fragile X syndrome/autism	Chen Zhang	Capital Medical University
Decision mechanism of human forebrain atlas at early embryonic stage	Xiaoqing Zhang	Tongji University
Engraftment of human cerebral organoids establish region‐specific projections in the adult mouse brain	Yan Liu	Nanjing Medical University
Alzheimer disease and neural regeneration	Qiang Liu	University of Science and Technology of China
The mechanism of TRPM2‐mediated dopaminergic neuronal death from C. Elegans to PD Patients iPSC	Wei Yang	Zhejiang University
AMPA receptor synaptic plasticity *in vivo*	Yong Zhang	Peking University
Transcriptional regulation in neural reprogramming and regeneration	Mengqing Xiang	Sun Yat‐sen University
Autism‐related protein ADNP regulates neural progenitor development and regeneration through WNT signal transduction	Yuhua Sun	Institute of Hydrobiology, Chinese Academy of Sciences
Concurrent Session 4 Organoids and Disease Models Co‐chair: Xiaoqun Wang, Lijian Hui		
Inner ear stem cell proliferation, *in situ* regeneration of inner ear hair cells, and establishment of inner ear organoids	Xiaolei Yin	Tongji University
Application of cerebral organoids to study human brain tumours	Shan Bian	Tongji University
A Novel 2D and 3D air–liquid interface organoids union culture system model colorectal cancer	Xia Wang	Tsinghua University
Developmental mechanism of stem cells	Ting Chen	National Institute of Biological Sciences, Beijing
Investigation of cancer stem cells and cancer organoids using single cell sequencing techniques	Pengcheng Bu	Institute of Biophysics, Chinese Academy of Sciences
Establishment of brain organoids	Yangfei Xiang	Shanghai Tech University
Synthesis of novel engineered cells in targeted recovery of blood‐brain barrier	Jianhong Zhu	Huashan Hospital
Human stem cell‐derived neurons repair circuits and restore neural function	Yuejun Chen	CAS Center for Excellence in Brain Science and Intelligence Technology
Application of hydrogel system in cell culture	Renjun Pei	Suzhou Institute of Nano‐Tech and Nano‐Bionics, Chinese Academy of Sciences
ERMAP, a novel B7 family‐related molecule	Min Su	Guizhou Medical University
Concurrent Session 5 Tissue and Organ Regeneration		
Co‐chair: Hongmei Wang, Tianqing Li		
Research and development of conductive tissue engineered neuron	Yumin Yang	Nantong University
Deciphering the secrets of how primates are formed	Hongmei Wang	Institute of Zoology, Chinese Academy of Sciences
3D Bioprinting, from organ models to tissue recovery	Yong He	Zhejiang University
Bionic manufacturing	Qi Gu	Institute of Zoology, Chinese Academy of Sciences
Xeno‐regeneration of pig organs	Jianguo Zhao	Institute of Zoology, Chinese Academy of Sciences
Novel techniques in establishing tissue engineering	Jianhua Qin	Dalian Institute of Chemical Physics, Chinese Academy of Sciences
Key scientific questions in cell therapy using islet xenotransplantation	Wei Wang	Xiangya Third Hospital Central South University
Vascular heart chambers and disease studies	Shijun Hu	Suzhou University
Application of iPS‐derived breast organs in breast regeneration and breast cancer studies	Ceshi Chen	Kunming Institute of Zoology, Chinese Academy of Sciences
Concurrent Session 6 Clinical Translation of Stem Cell Research		
Co‐chair: Tongbiao Zhao, Yali Hu		
Studies of safety evaluation non‐clinical stem cell therapy products	Yan Huo	National Institutes for Food Drug Control
Quality and capability of stem cell testing laboratory	Jing Lv	China National Accreditation Service for Conformity Assessment
Progress and FAQs of registered stem cell clinical studies	Zhaohui Wu	China Medicinal Biotechnology Association
Ethics and regulatory studies on stem cell clinical transformation	Yaojin Peng	Institute for Stem Cell and Regeneration, Chinese Academy of Sciences
hESC‐derived stem cell drug development and its application in COVID‐19 patients	Jie Hao	Institute of Zoology, Chinese Academy of Sciences
Application studies of hair follicle stem cell in tissue regeneration, organ reconstruction and gene therapy	Jinyu Liu	Jilin University
Seed cell selection and internal quality standards development in cell therapies	Bin Wang	Nanjing University Medical School Affiliated Drum Tower Hospital
Efficacy evaluation and preliminary investigation on mechanism in mesenchymal stem cell treatment in acute respiratory distress syndrome	Peng Xiang	Sun Yat‐sen University

## 7 SPECIAL SESSION ON INTERNATIONAL COLLABORATION

The special session of the Chinese International Stem Cell Network (CISCN) was organized as a virtual conference and hosted by Prof Glyn Stacey (supported by CAS President's International Fellowship as a Special Expert). This special session aimed to encourage collaboration on stem cell standard development. Collaborating scientists with the Institute for Stem Cell and Regeneration/Institute of Zoology, Chinese Academy of Sciences were invited to participate in this special session. CSSCR president, academician Weizhi Ji introduced the Chinese Society for Stem Cell Research and shared the ongoing progress of this congress. Prof Andreas Kurtz of Fraunhofer‐IBMT (Berlin, Germany) delivered a presentation on hPSC data management and sharing. Prof Tongbiao Zhao (Institute for Stem Cell and Regeneration, CAS) also gave a lecture entitled ‘Integrated Efforts on Stem Cell Standard Development and Open Sharing’.

## 8 CO‐ORGANIZED SEMINARS

The congress provided 4 co‐organized luncheon seminars. The pre‐meeting luncheon seminar *Cell Proliferation* Forum was sponsored by the CSSCR flagship journal *Cell Proliferation*. Two presentations were invited to report their work that had just been published in a special issue of *Cell Proliferation* on Stem Cell Therapy for COVID‐19 and other Major Diseases. Dr Jianyuan Wu (on behalf of Prof Xinghuan Wang, the President of Zhongnan Hospital, Wuhan University) presented ‘Safety and Feasibility of Umbilical Cord Mesenchymal Stem Cells in Patients with COVID‐19 Pneumonia: A Pilot Study)^2^’. Dr Liu Wang from the National Stem Cell Resource Center in her talk entitled ‘Using CAStem in Treatment for Covid‐19 Pulmonary Fibrosis^3^’ disclosed alternative approaches to get anti‐inflammatory cells. She shared the pre‐clinical results using the innovative stem cell‐derived medicinal drug ‘CAStem™’ (immunity‐ and matrix‐regulatory cells) to treat pulmonary injury and fibrosis. Associate editor, Prof Shiqiang Huang from the Institute for Stem Cell and Regeneration/Institute of Zoology, Chinese Academy of Sciences shared the peer review process of Cell Proliferation. Dr Tina Wang, senior journal publishing manager from Wiley publishing group presented a lecture ‘Getting Published in *Cell Proliferation*’. Dr Wang shared some tips for manuscript submission and major concerns on publishing ethics.

## 9 YOUTH FORUM

The ‘youth forum’ for young scientists has long been the traditional special event of CSSCR Congress. A total of 10 junior scientists were invited to present on diverse topics including cell fate decision, maintenance and regulation of pluripotency, RNA switch‐based gene therapies, stem cell manufacturing etc. The youth forum was chaired by Prof Yangming Wang (Peking University) and Prof Jiekai Chen (Guangzhou Institutes of Biomedicine and Health, Chinese Academy of Sciences). Prof Xiangying Li (Peking University) delivered a speech entitled ‘Regulation of Chromatin Landscape during Erythropoiesis’. Prof An Zeng (CAS Center for Excellence in Molecular Cell Sciences) talked on ‘Maintenance and Regulation of Adult Pluripotency during Planarian Regeneration’. Dr Le Sun (Institute of Biophysics, Chinese Academy of Sciences) and colleagues are applying *in vivo* recording with single cell sequencing techniques to reveal molecular properties of mouse V1.^4^ Prof Hengyou Weng shared the important role of epi‐transcriptomic N6‐methyladenine (m6A) mRNA modification in stem cell fate decision and maintenance. Prof Guocai Zhong (Shenzhen Bay Laboratory) gave a lecture on ‘Technical Investigation on RNA Switch‐based Gene Therapies’. Dr Ying Zhang from Nuwacell shared their prospects on an iPSC bank of ‘super donor’ for Chinese population. Dr Si Wang (Institute of Zoology, Chinese Academy of Sciences) presented her work on plotting a single‐cell transcriptomic atlas of primate ovarian ageing.^5^ Prof Chuan Ye (The Affiliated Hospital of Guizhou Medical University) overviewed stem cell biomanufacturing techniques. Dr Jiayu Chen shared the parallel analysis of epigenetic mark H3K9ac to reveal Dux‐dependent 2‐cell genome activation deficiency in somatic cell nuclear transfer embryos.^6^ Dr Jian Yang (Tongji University) and his colleagues talked about R&D of stem cell‐based pacemaker.

## 10 THE AWARDS CEREMONY

The CSSCR congress confers awards on innovation, translational science, popularization of science, social welfare, young scientist and distinguished scientist to recognize significant and original scientific contributions to the science of stem cell research and clinical application. The awardee of the Outstanding Contribution Award of this year was Xiuwu Bian, for his outstanding scientific contribution to tumour stem cells and COVID‐19 pathology, as well as his continued support and direct efforts to boost CSSCR. A Special Contribution Award was set this year to recognize the outstanding scientists and team in the fight against the COVID‐19 pandemic. The award titles and awardees are listed in Table 2 (see end of this document).

**TABLE 2:** CSSCR awards conferred in this congress

Award title	Awardee	Affiliation
CSSCR‐ZEISS Innovation Award	Tianqing Li	Kunming University of Science and Technology
	Wei Li	Institute of Zoology, Chinese Academy of Sciences
	Yi Zeng	CAS Center for Excellence in Molecular Cell Sciences
	Jianhong Zhu	Fudan University
CSSCR Junior PI Award	Tao Tan	Kunming University of Science and Technology
	Jing Qu	Institute of Zoology, Chinese Academy of Sciences
	Qian Wu	Institute of Biophysics, Chinese Academy of Sciences
Obsessed with science prize for the popularization of science	Tianda Li	Institute of Zoology, Chinese Academy of Sciences
	Jing Lu	Institute of Zoology, Chinese Academy of Sciences
	Yizhu Wang	Institute of Zoology, Chinese Academy of Sciences
Translational science award	Xiaojun Huang	Peking University
Outstanding contribution award	Xiuwu Bian	Third Military Medical University (PLA)
Special contribution award	Jie Hao	National Stem Cell Resource Center
	The CAStem™ Clinical Research Team (group awardee)	
The social welfare group award	The Standard Working Group of CSSCR	
	The Local Organizing Committee of the 9th Congress of the CSSCR	

## 11 CONCLUDING REMARKS

The 10th Congress of the Chinese Society for Stem Cell Research was held at a very special time, when the world was threatened by the COVID‐19 pandemic. Regardless, the 1200 participants including 700 off‐line and 500 on‐line have had a very successful event. Aligned with its theme ‘However difficult it might appear; the challenges will be overcome’, the congress offered diverse and cutting‐edge topics on stem cell research and clinical studies, standards development, ethics and regulatory policies. Altogether with special sessions on international collaboration and journal publication, we hope that it provided opportunities to promote additional advancements in this exciting field and better engagement of Chinese scientists with the global innovation network. We are grateful to the participants, co‐organizing institutions, members of the scientific advisory board and local organizing committee for their efforts in this event.

## 12 RECENT CSSCR EFFORTS IN STANDARDIZATION FOR STEM CELL RESEARCH

A concurrent special event on stem cell standardization was organized by the standards working group of CSSCR. This working group was established in 2016. The main responsibilities of the standards working group include standard development, standard information dissemination, standard information updating and tracking, standardization research, standard information resource platform construction, standard publicity and training etc., and are committed to promoting the field of cell biology for the standardization process from foundation to transformation. The standards working group recently revised the standards ‘General requirements for stem cells’ and ‘Requirements for Human Embryonic Stem Cells’ on 30 April 2020, which were released in 2017 and 2019 respectively. With help of the CAS PIFI expert Prof Glyn Stacey, the translated version of the standards ‘General requirements for stem cells’^7^ and ‘Requirements for Human Embryonic Stem Cells’^8^ were published in Cell Proliferation in December 2020. Six standards projects were initiated at the standards working group meeting during the 10th Congress of CSSCR, including (1) Guidelines for ethical review of human stem cell research; (2) Human neural stem cell; (3) Method for detecting residual human pluripotent stem cells in differentiated cells; (4) Flow cytometry—Guidelines for the detection of stem cell marker proteins; (5) General rules for determination of cell viability assay; (6) Detection method of IDO1 bioactivity of mesenchymal stromal cells.

## CONFLICT OF INTEREST

The authors declare that they have no conflict of interest.

## AUTHOR CONTRIBUTIONS

JC manuscript writing. BH, WJ speaker at conference, provision of content for meeting, final manuscript approvers.

## Data Availability

Data sharing is not applicable to this article as no new data were created or analyzed in this study.
